# Intrinsic apoptotic pathway activation increases response to anti-estrogens in luminal breast cancers

**DOI:** 10.1038/s41419-017-0072-x

**Published:** 2018-01-17

**Authors:** Michelle M. Williams, Linus Lee, Thomas Werfel, Meghan M. Morrison Joly, Donna J. Hicks, Bushra Rahman, David Elion, Courtney McKernan, Violeta Sanchez, Monica V. Estrada, Suleiman Massarweh, Richard Elledge, Craig Duvall, Rebecca S. Cook

**Affiliations:** 1 Department of Cancer Biology, Vanderbilt University School of Medicine, Nashville, TN USA; 2 Department of Biomedical Engineering, Vanderbilt University School of Engineering, Nashville, TN USA; 3 Department of Medicine, Vanderbilt University Medical Center, Nashville, TN USA; 4 Department of Medicine, Stanford University School of Medicine, Stanford, CA USA; 5 Cancer Therapy and Research Center, University of Texas Health Science Center, San Antonio, TX USA; 6 The Vanderbilt-Ingram Cancer Center, Vanderbilt University Medical Center, Nashville, TN 37232 USA

## Abstract

Estrogen receptor-α positive (ERα+) breast cancer accounts for approximately 70–80% of the nearly 25,0000 new cases of breast cancer diagnosed in the US each year. Endocrine-targeted therapies (those that block ERα activity) serve as the first line of treatment in most cases. Despite the proven benefit of endocrine therapies, however, ERα+ breast tumors can develop resistance to endocrine therapy, causing disease progression or relapse, particularly in the metastatic setting. Anti-apoptotic Bcl-2 family proteins enhance breast tumor cell survival, often promoting resistance to targeted therapies, including endocrine therapies. Herein, we investigated whether blockade of anti-apoptotic Bcl-2 family proteins could sensitize luminal breast cancers to anti-estrogen treatment. We used long-term estrogen deprivation (LTED) of human ERα+ breast cancer cell lines, an established model of sustained treatment with and acquired resistance to aromatase inhibitors (AIs), in combination with Bcl-2/Bcl-xL inhibition (ABT-263), finding that ABT-263 induced only limited tumor cell killing in LTED-selected cells in culture and in vivo. Interestingly, expression and activity of the Bcl-2-related factor Mcl-1 was increased in LTED cells. Genetic Mcl-1 ablation induced apoptosis in LTED-selected cells, and potently increased their sensitivity to ABT-263. Increased expression and activity of Mcl-1 was similarly seen in clinical breast tumor specimens treated with AI + the selective estrogen receptor downregulator fulvestrant. Delivery of Mcl-1 siRNA loaded into polymeric nanoparticles (MCL1 si-NPs) decreased Mcl-1 expression in LTED-selected and fulvestrant-treated cells, increasing tumor cell death and blocking tumor cell growth. These findings suggest that Mcl-1 upregulation in response to anti-estrogen treatment enhances tumor cell survival, decreasing response to therapeutic treatments. Therefore, strategies blocking Mcl-1 expression or activity used in combination with endocrine therapies would enhance tumor cell death.

## Introduction

The American Cancer Society estimated that approximately 25,0000 women were diagnosed with breast cancer in 2016 in the United States alone^1^. The most frequently diagnosed clinical breast cancers are those expressing estrogen receptor-α (ERα), a nuclear receptor driving cell cycle progression. ERα+ breast cancers are treated with targeted inhibitors that block ERα signaling, including selective ERα modulators (SERMS, e.g., tamoxifen), selective ERα downregulators (SERDs, e.g., fulvestrant) and AIs that decrease circulating estrogen in post-menopausal women. Although these treatments are successful for a large number of breast cancer patients, 15–30% display de novo or acquired resistance to anti-estrogens (reviewed in refs.^2, 3^). Given the number of new diagnoses, and the numerous breast cancer-related deaths caused by anti-estrogen resistance each year, there is a need to identify molecular vulnerabilities in ERα+ tumors for preventing or overcoming anti-estrogen resistance.

Resistance to many cancer treatments relies on evasion of cell death^4^, often caused by expression or activity of anti-apoptotic Bcl-2 family proteins (Bcl-A1, Bcl-2, Bcl-xL, Bcl-w, and Mcl-1). These factors prevent Bak/Bax oligomerization and pore formation in the outer mitochondrial membrane (as reviewed in refs.^5, 6^) by binding directly to Bak or Bax^7^, or to Bim, an activator of Bak/Bax oligomerization^8^. ERα+ breast cancers frequently overexpress anti-apoptotic Bcl-2, Bcl-xL, and Mcl-1^9–12^. Bcl-2 and Bcl-xL are further elevated upon anti-estrogen treatment^13–16^, suggesting that ERα+ breast cancers may use anti-apoptotic Bcl-2 family members to drive cell survival and treatment resistance^17, 18^.

Anti-estrogens are often cytostatic^19^, halting cell proliferation without activating apoptosis. Survival of tumor cells during treatment would increase the likelihood of recurrence upon treatment withdraw, and may enforce treatment resistance, suggesting that blockade of anti-apoptotic Bcl-2 proteins in combination with anti-estrogens may decrease recurrence and/or resistance in ERα+ breast cancers. This idea has been tested using small molecular weight inhibitors known as ‘BH3-mimetics,’ designed to bind anti-apoptotic Bcl-2 proteins within their BH3-interaction motif, preventing association with pro-apoptotic proteins Bax and Bim^20^. Although Bcl-2/Bcl-xL inhibition using the BH3-mimetic ABT-737, or Bcl-2 specific inhibition, using the BH3-mimetic ABT-199, had little activity as single agents in breast cancers, their combination with tamoxifen resulted in tumor regression in some, but not all, patient-derived ERα+ breast cancer xenografts tested^13^, supporting a role for Bcl-2 in endocrine resistance. Other studies, however, show that *BCL2* is an ERα transcriptional target, and is decreased in tamoxifen-treated and tamoxifen-resistant xenografts^21^. These conflicting results require continued exploration of Bcl-2 family members ERα+ breast cancers.

To investigate this, we used long-term estrogen deprivation (LTED) to model treatment with and acquired resistance to AIs in human luminal breast cancer cell lines. We found that Bcl-2/Bcl-xL inhibition did not increase cell death in LTED-selected cells. However, Mcl-1 expression and activity were upregulated upon estrogen deprivation, as well as in response to fulvestrant. The recent development of Mcl-1-specific BH3-mimetics is allowing preclinical testing of Mcl-1 inhibition in some cancers^22–24^, leading in some cases to clinical trials^25^. However, preclinical and clinical testing of Mcl-1 blockade in combination with endocrine inhibition in ERα+ breast cancers is not fully explored. Targeted inhibition of Mcl-1 in ERα+ breast cancers using Mcl-1 siRNA encapsulated in polymeric nanoparticles (si-NPs) increased tumor cell killing in LTED-selected cells and in fulvestrant-treated cells, demonstrating that anti-estrogens prime ERα+ cells for targeted Mcl-1 inhibition.

## Results

### Long-term estrogen deprivation does not sensitize cells to Bcl-2/Bcl-xL inhibition

**Fig. 1:**
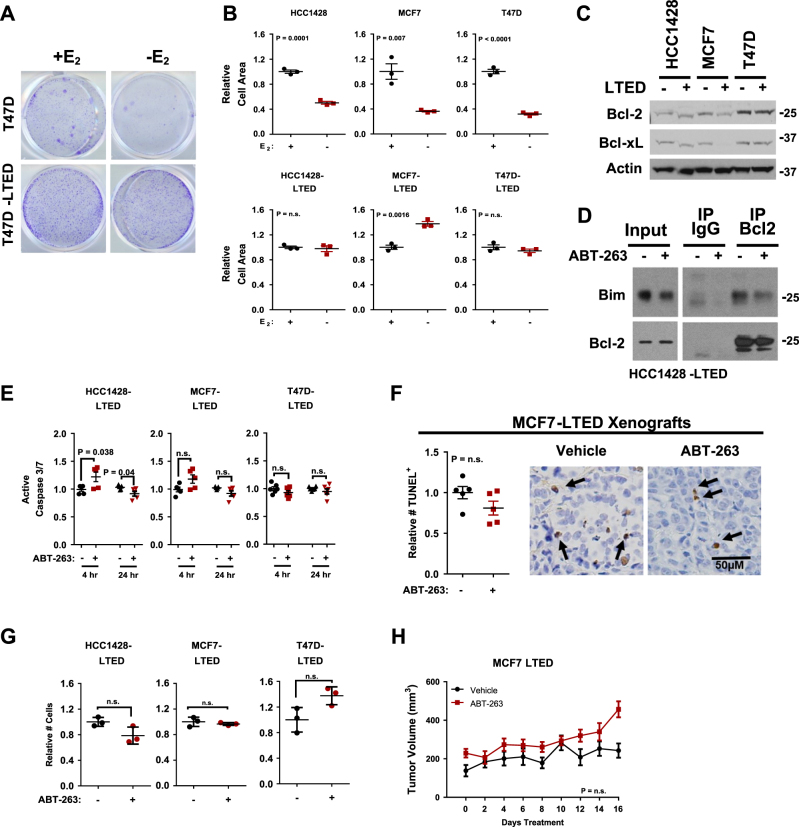
LTED-selected cells remain insensitive to Bcl-2/Bcl-xL inhibition. **a**,**b** Crystal violet staining completed on parental and LTED-selected cells grown ± estrogen (E_2_) for 7 days. Representative images are shown (**a**). Stained cell area/well was quantitated using the ImageJ software then used to quantitate average cell area (**b**). *N* = 3, each measured in triplicate. Student’s unpaired two-tailed *t*-test. **c** Whole cell lysates harvested from parental or LTED-selected cells were assessed by western analysis using antibodies indicated at left, molecular weight in kiloDaltons. **d** Western analysis of whole cell lysate (input) or Bcl-2 IPs from whole cell lysate harvested from HCC1428-LTED cells treated 2 h with ABT-263 (1.0 µm) was performed using antibodies shown at left, molecular weight in kiloDaltons. **e** Caspase-3/7 activity was evaluated in cells treated 4 and 24 h with 1.0 µm ABT-263 (Caspase-3/7 Glo). Data points are shown relative to the average luminescence measured in dimethyl sulfoxide (DMSO)-treated cells, set equal to a value of 1. Luminescence for the average of five separate experiments, *N* = 5 repeats of three experimental replicates each. Student’s unpaired two-tailed *t*-test. **f** terminal dUTP nick end labeling (TUNEL) analysis of MCF7-LTED tumors grown in mice treated 16 days with daily ABT-263 (20 mg/kg) or vehicle control and harvested 1 h after final treatment. Left: representative images (×200), Right: average TUNEL^+^ cells/field, *N* = 5 tumors, 6 fields per tumor. Arrows denote TUNEL^+^ cells. **g** Total number of cells/well were counted after treatment with DMSO or ABT-263 (1.0 µm) for 48 h. Data represent average of three separate experiments, *N* = 3, Student’s unpaired two-tailed *t*-test. **h** Average tumor volume of MCF7-LTED xenografts treated for 16 days with daily ABT-263 (20 mg/kg). For **b**, **f**−**h** error bars represent standard error

To model long-term treatment with AIs, three human ERα+ breast cancer cell lines, HCC1428, MCF7, and T47D, were cultured under estrogen deprivation for 3–6 months as described in previous studies^26^. While parental cells, which are maintained in estrogen-replete conditions, exhibited growth inhibition upon acute estrogen withdraw, LTED-selected cells grew at a similar or accelerated rate in estrogen-depleted media as compared to their growth in estrogen-replete conditions (Fig. 1a, b). Since previous studies show that anti-apoptotic Bcl-2 family members Bcl-2 and Bcl-xL promote cell survival in tamoxifen-treated breast cancers^13^, we measured expression of Bcl-2 and Bcl-xL in parental and LTED-selected breast cancer cells, finding similar levels of Bcl-2 and Bcl-xL in LTED-selected HCC1428 and T47D cells as compared to their parental counterpoints cultured in estrogen-replete media (Fig. 1c). LTED-selected MCF7 cells expressed less Bcl-2 and less Bcl-xL as compared to parental counterparts. Interestingly, parental MCF7 cells upregulated Bcl-xL alone upon acute estrogen deprivation (3 days and 7 days), while parental HCC1428 cells upregulated Bcl-2 alone upon acute estrogen deprivation (Supplemental Figure S1A). These results suggest that, although acute estrogen deprivation may transiently enhance Bcl-2 and/or Bcl-xL, LTED does not.

Despite no obvious Bcl-2 and/or Bcl-xL protein upregulation in LTED-selected cells, it is possible that the anti-apoptotic activities of Bcl-2 and/or Bcl-xL (i.e. sequestration of pro-apoptotic Bim or Bax) support cell survival and therapeutic resistance in this setting. To test this, activity of Bcl-2 and Bcl-xL was blocked in LTED-selected cells using the BH3-mimetic ABT-263 (1.0 µm). Bcl-2 immuno-precipitates assessed by western analysis for Bim confirmed that ABT-263 reduced Bcl-2/Bim interactions (Fig. 1d and Supplemental Figure S1B). Previous reports show that ABT-263 treatment of HCC1428, MCF7, and T47D cells induced caspase-3/7 activity after 4 h^12^, similar to observations in other cell lines^27^. In contrast, ABT-263 did not increased caspase-3/7 activity in MCF7-LTED and T47D-LTED cells at 4 or 24 h (Fig. 1e), while HCC1428-LTED cells responded to ABT-263 with a subtle increase in caspase-3/7 activity at 4 h only. These results were confirmed in vivo using MCF7-LTED xenografts treated daily with ABT-263 (20 mg/kg), which did not increase tumor cell death as assessed by terminal dUTP nick end labeling (TUNEL) analysis (Fig. 1f). Consistent with these results, cell growth (total cell number) of LTED-selected cells was not affected by 48 h treatment with ABT-263 (Fig. 1g). Similarly, MCF7-LTED xenograft growth (tumor volume) was not affected by daily ABT-263 treatment (Fig. 1h), even though Bim/Bcl-2 interactions were disrupted by ABT-263 (Supplemental Fig. 2a). Tumor cell proliferation also was unaffected by ABT-263 (Supplemental Fig. 2b). These data suggest that LTED, such what is achieved by AIs, may not prime ERα+ breast cancer cells for Bcl-2/Bcl-xL targeting.

### Mcl-1 expression increases under long-term estrogen deprivation

**Fig. 2:**
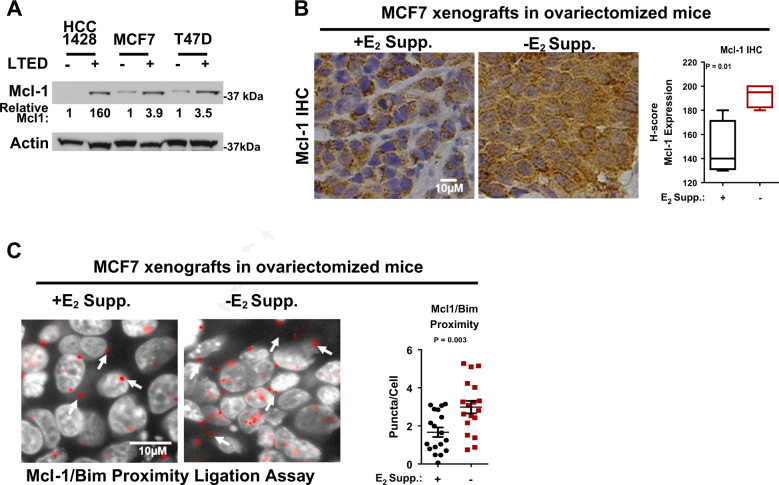
Mcl-1 upregulation in response to LTED. **a** Whole cell lysates were assessed by western analysis using antibodies shown at right. ImageJ was used for densitometry analysis to assess Mcl-1 levels (corrected for actin levels) shown relative to levels seen in the respective parental cell line set to a value of 1. **b**,**c** MCF7 xenografts were grown for 42 days in ovariectomized mice with or without estrogen supplementation. Mcl-1 immunohistochemistry with representative images at ×400 are shown in the left panels (**b**). Quantitation (right panel) of Mcl-1 IHC performed by a pathologist is represented as an H-score (a semi-quantitative scoring system that factors staining intensity and percentage of cells positive for staining). Student’s unpaired two tailed *t*-test, error bars represent min to max. **c** depicts representative images of Mcl-1/Bim PLA (×630), gray = DAPI, red = Mcl-1/Bim proximity (denoted by arrows). Quantitation of Mcl-1/Bim proximity as puncta/cell for six fields per tumor. Student’s unpaired two tailed *t*-test, error bars represent standard error

Previous studies demonstrated that Mcl-1, a related anti-apoptotic Bcl-2 protein, is upregulated upon Bcl-2/Bcl-xL inhibition, resulting in resistance to ABT-263^22, 28–32^. In fact, Mcl-1 expression levels correlated inversely with sensitivity to ABT-263 in untreated ERα+ breast cancer cells^12^. Interestingly, Mcl-1 levels were elevated in HCC1428-LTED, MCF7-LTED, and T47D-LTED cells as compared to parental counterparts (Fig. 2a). We also measured relative *BIK* and *PUMA* transcript levels in cells selected for LTED resistance, based on previous reports that anti-estrogen treatments result in upregulation of Bik, but not Puma^33, 34^. Similar to previous reports, we found increased *BIK* in MCF7-LTED and T47D-LTED over what was seen in MCF7 parental and T47D parental cell lines, respectively, while *PUMA* transcript levels were unaltered in LTED cells as compared to parental counterparts (Supplemental Figure 3a). Although the trend towards *BIK* upregulation in HCC1428-LTED cells over parental HCC1428 cells did not reach statistical significance, together these findings suggest that ERα+ breast cancer cells respond to sustained estrogen deprivation through *BIK* and Mcl-1 upregulation.^33, 34^ Mcl-1 upregulation upon sustained estrogen deprivation was confirmed by immunohistochemical staining in MCF7 xenografts grown in ovariectomized hosts (thus, estrogen deprived for 42 days), revealing increased Mcl-1 staining intensity, as compared to MCF7 xenografts grown in ovariectomized hosts supplemented with 42-day release estrogen pellets (Fig. 2b, and Supplemental Figure S3b). We used Proximity Ligation Assay (PLA^35^), a technique measuring protein−protein interactions in situ, to quantitate Mcl-1 activity (Mcl-1/Bim interactions) in MCF7 xenografts. Fluorescent puncta, indicating Bim/Mcl-1 interaction, were increased in tumors grown in ovariectomized mice without estrogen supplementation (Fig. 2c), suggesting that, despite upregulation of pro-apoptotic forces such as *BIK*, increased Mcl-1 expression and activity upon sustained estrogen deprivation also increase, perhaps counteracting *BIK* upregulation and promoting cell survival.

### Fulvestrant primes luminal breast cancers for Mcl-1 targeting

**Fig. 3:**
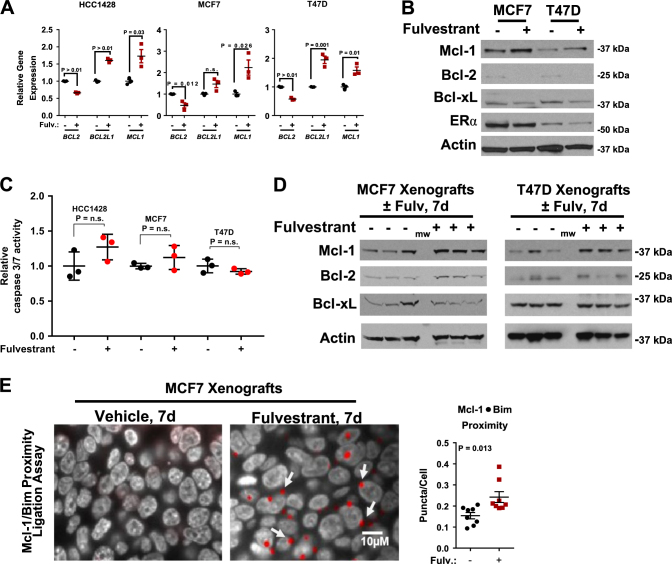
Selective estrogen receptor downregulators increase Mcl-1 expression and activity. **a**,**b** RNA (**a**) or whole cell lysates (**b)** were harvested from cells treated with 1 µm fulvestrant for 24 or 6 h, respectively. mRNA expression was analyzed by RT-qPCR and protein expression was analyzed using antibodies denoted on the left. **c** Caspase -3/7 activity was assessed in cells treated 24 h with fulvestrant (1 µm) or DMSO. Values shown are the average of two experimental replicates, while the midlines are the average of *N* = 3 experiments. **d**,**e** MCF7 and T47D xenografts grown in mice with intact ovaries were treated with fulvestrant once by intraperitoneal injection and harvested after 7 days. **d** Whole tumor lysates were assessed by western analysis using antibodies shown at left. **e** PLA completed on FFPE tissues. Left: representative images (×630), gray = DAPI, red = Mcl-1/Bim proximity (arrows denote Mcl-1/Bim interactions). Right: quantitation represents the average number of puncta/cell for six images per tumor, *N* = 8. Student’s unpaired two-tailed *t*-test, error bars represent standard error

We next examined Bcl-2, Bcl-xL, and Mcl-1 expression changes in response to fulvestrant, a SERD that blocks estrogen binding to ERα and induces ERα degradation. Parental HCC1428, MCF7, and T47D cells treated 24 h with fulvestrant (1.0 µm) showed decreased *BCL2* transcripts (Fig. 3a), while *BCL2L1* (Bcl-xL) levels increased in HCC1428 and T47D cells. *MCL1* transcripts were elevated in all three fulvestrant-treated cell lines. Mcl-1 protein expression in parental MCF7 and T47D cells increased upon fulvestrant treatment, while Bcl-2, detected at very low levels in MCF7 and T47D cells, diminished further in response to fulvestrant (Fig. 3b), as did Bcl-xL. Importantly, fulvestrant did not induce significant levels of caspase-3/7 activity in cultured HCC1428, MCF7, or T47D cells (Fig. 3c). These findings were verified in vivo using MCF7 and T47D xenografts treated 7 days with fulvestrant, revealing fulvestrant-mediated ERα downregulation (Supplemental Figure S4) and Mcl-1 upregulation (Fig. 3e). Further, PLA-mediated detection of Mcl-1/Bim interactions demonstrated that fulvestrant increased Mcl-1 activity (Fig. 3d). Bcl-2 levels increased in fulvestrant-treated MCF7 (but not T47D) tumors, while Bcl-xL showed relatively little change (Fig. 3d).

### Genetic targeting of *MCL1* increases apoptosis in LTED-selected breast cancer cells

**Fig. 4:**
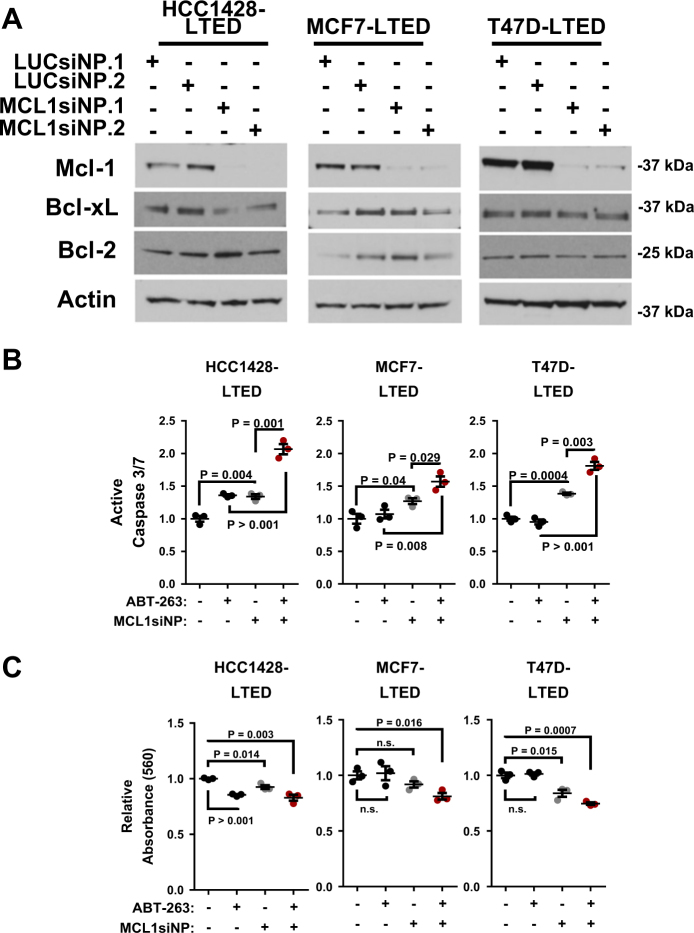
Mcl-1 inhibition restores sensitivity of LTED breast cancers to ABT-263. **a** Whole cell lysates from LTED-selected cells were assessed by western analysis for antibodies indicated at left after treatment with siNPs loaded with 50 nm siControl or siMcl1 for 48 h. **b**,**c** LTED-selected cells were treated for 48 h with 50 nm si-NP. 1.0 µm ABT-263 was added for the final 4 h. Caspase-3/7 activity was measured (Caspase-3/7 Glo, **b**) and used to calculate average luminescence, relative to values obtained for LUC si-NP-treated cells with DMSO treatment, set to a value of 1. *N* = 3, each assessed in duplicate. Student’s unpaired two-tailed *t*-test, error bars represents standard error. Total protein was quantitated using an SRB assay (**c**), relative to values obtained for LUC si-NP-treated cells with DMSO treatment, set to a value of 1. *N* = 3, each assessed in duplicate. Student’s unpaired two-tailed *t*-test, error bars represent standard error

We delivered siRNA sequences directed against Mcl-1 to LTED-selected ERα+ breast cancer cells as an experimental approach at targeted and highly selective Mcl-1 inhibition. As a control, we used luciferase-directed siRNA sequences. Delivery of siRNA sequences was achieved using polymeric siRNA nanoparticles (si-NPs), including MCL1 si-NPs and LUC si-NPs, respectively. These recently developed nanocarriers enable effective siRNA delivery/activity in cultured cells due to high cell uptake and efficient endosomal escape. Also, unlike other off-the-shelf agents, these NPs can be directly translated into future in vivo studies due to sufficient serum stability that enables i.v. injection and biodistribution/bioactivity within breast tumors^36^. Cells were assessed for Mcl-1 knockdown by western analysis 48 h after treatment with si-NPs, demonstrating that MCL1 si-NPs decreased Mcl-1 protein expression in all three LTED cells (Fig. 4a). Importantly, Bcl-2 and Bcl-xL levels remained unchanged despite Mcl-1 knockdown in MCL1 si-NP-treated cells, confirming target sequence specificity, and ruling out acute compensatory upregulation of Bcl-2 and Bcl-xL upon Mcl-1 gene targeting.

HCC1428-LTED, MCF7-LTED, and T47D-LTED cells treated with MCL1 si-NPs displayed increased caspase-3/7 activity as compared to cells treated with LUC si-NPs (Fig. 4b). Caspase-3/7 activity was increased further in LTED-selected cells when MCL1 si-NPs were used in combination with ABT-263. MCL1 si-NPs decreased growth of LTED-selected cells as compared to LUC si-NPs (Fig. 4c), and increased growth inhibition in response to ABT-263.

### Genetic targeting of *MCL1* increases apoptosis in Fulvestrant-treated breast cancer cells

**Fig. 5:**
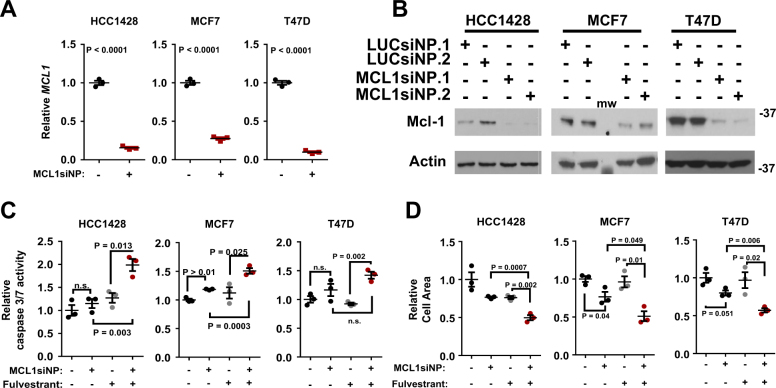
Mcl-1 inhibition sensitizes ERα+ breast cancers to fulvestrant. Cells were treated with siNPs loaded with 100 nm siControl or siMcl1 for 24 h, and cultured for an additional 24 h in growth media (**a**,**b**) or in growth media with and without 1 µm fulvestrant (**c**,**d)**. **a** *MCL1* expression was assessed in total RNA by RT-qPCR, *N* = 3 assessed in triplicate. **b** Whole cell lysates were assessed by western analysis using antibodies shown at left, molecular weight kiloDaltons. **c** Caspase-3/7 activity was measured (Caspase-3/7 Glo) in duplicate, *N* = 3 (**c**). **d** Cells were treated with siNP (100 nm siLUC or siMCL1) for 24 h then cultured 7 days in growth media supplemented with or without 1 µm fulvestrant. Cells were stained with crystal violet, and fluorescent cell area was scanned. Total fluorescent area was used as a measure of total cellular area. Quantitation represents average area stained with crystal violet per well, *N* = 3–4, each conducted in triplicate. Student’s unpaired two-tailed *t*-test. For **a**, **c**, **d** error bars represent standard error

We next examined the impact of MCL1 si-NPs in parental ERα+ breast cancer treated with fulvestrant, first verifying Mcl-1 knockdown in parental HCC1428, MCF7, and T47D cells at 48 h post-treatment, revealing 70–90% fewer *MCL1* transcripts as compared to cells treated with LUC si-NPs (Fig. 5a). These results were confirmed by western analysis at this same timepoint (Fig. 5b). We next measured caspase-3/7 activity in ERα+ breast cancer cells 48 h after treatment with MCL1 si-NPs. Unlike what was seen in LTED-selected cells, which exhibited increased cell death upon knockdown of Mcl-1, two of three parental cells did not increase caspase-3/7 activity in response to MCL1 si-NPs (Fig. 5c). Consistent with previous reports, fulvestrant as a single agent also did not induce caspase-3/7 activity in HCC1428, MCF7, or T47D cells. However, the MCL1 si-NPs combined with fulvestrant robustly increased caspase-3/7 activity in HCC1428, MCF7, or T47D cells. Interestingly, MCL1 si-NPs alone decreased growth of parental MCF7, but not HCC1428 or T47D cells, over 7 days (Fig. 5d). As expected, fulvestrant decreased growth of ERα+ cell lines. However, the combination of fulvestrant + MCL1 si-NPs decreased growth of HCC1428, MCF7, and T47D to a greater extent than either agent alone.

### Mcl-1 activity increases clinically, highlighting a molecular vulnerability

**Fig. 6:**
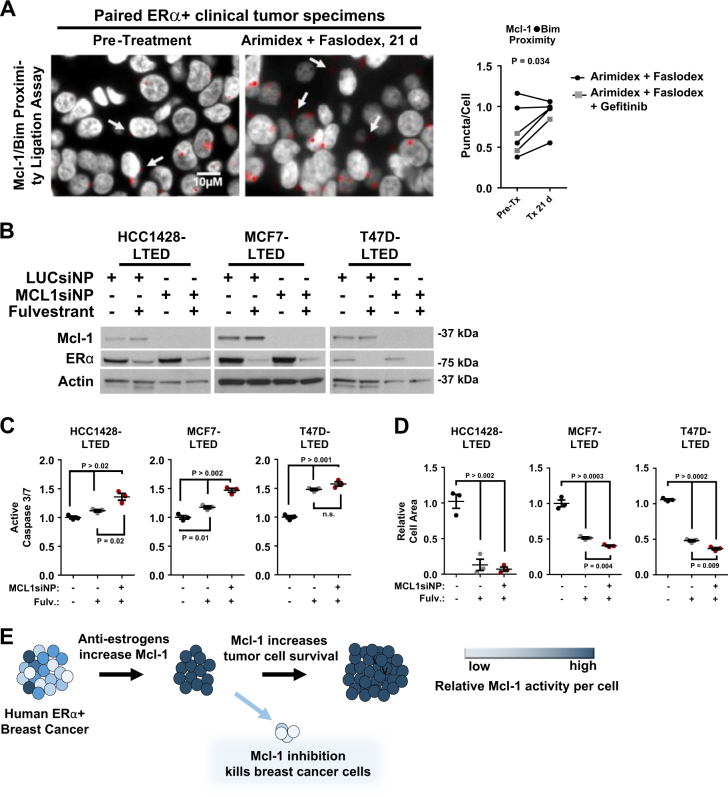
Mcl-1 activity increases in patients, highlighting a molecular vulnerability. **a** PLA to detect Mcl-1/Bim interactions was completed on de-identified clinical breast tumor specimens collect pre-treatment (Pre-Tx) and 21 days post treatment (Tx 21 days) with fulvestrant/Anastrozole ± gefitinib. Left: representative images (×630), gray = DAPI, red = Mcl-1/Bim proximity (arrows denote Mcl-1/Bim interactions). Right: quantitation represents the number of puncta/cell for each specimen. Student’s paired one-tailed *t*-test. Whole cell lysates from LTED-selected cells were assessed by western analysis for antibodies indicated at left after treatment with siNPs loaded with 100 nm siControl or siMcl1 for 48 h. **b**–**d** LTED-selected cells were treated for 48 h with 100 nm si-NP. For the final 24 h LTED growth media was replenished ±1.0 µm fulvestrant. **b** represents whole cell lysates assessed by western analysis using antibodies shown at right. **c** represents Caspase-3/7 activity (average luminescence) relative to LUC si-NP-treated cells with DMSO control, set to a value of 1. *N* = 3–4, conducted in triplicate. Student’s unpaired two-tailed *t*-test, error bars represent standard error. **d** LTED-selected cells were treated with siNP (100 nm siControl or siMcl1) for 24 h then cultured 7 days in growth media supplemented with or without 1.0 µm fulvestrant. Cells were stained with crystal violet, and fluorescent cells were then quantitated by scanning (Odyssey). Total fluorescent area was used as a measure of cell number. Quantitation represents average area stained with crystal violet per well, *N* = 2, each conducted in triplicate. **e** Schematic representation demonstrating that ERα+ breast cancers treated with anti-estrogens rapidly upregulate Mcl-1 expression, resulting in increased cell survival, decreasing treatment responses. However, we propose that Mcl-1 inhibition in combination with anti-estrogens will improve treatment response through increased tumor cell killing

To assess whether Mcl-1 is an essential tumor cell survival factor during a standard of care treatment regimen (i.e. first-line treatment with AIs, followed by second-line treatment with fulvestrant upon AI resistance), we first analyzed matched pairs of clinical breast tumor specimens from patients with ERα+ breast cancers for Mcl-1/Bim interactions by PLA. Breast tumor biopsies were collected from post-menopausal women with a new diagnosis of ERα+ breast cancer as part of a single-stage, single institution phase II neoadjuvant trial^37, 38^. Tumors were biopsied 24 h prior to any treatment (day 0). Patients were treated with a single fulvestrant dose (Faslodex, 250 mg) followed by daily treatment with the AI anastrozole (Arimidex,1 mg daily). In some cases, patients also received daily treatment with the epidermal growth factor receptor (EGFR) inhibitor gefitinib. Tumor biopsies were collected again on treatment day 21. These studies revealed that Bim interactions with Mcl-1 were increased in post-treatment specimens (day 21) as compared to those collected prior to treatment (day 0, Fig. 6a), consistent with the results shown here in animal models. Although the inclusions of the EGFR kinase inhibitor gefitinib is a caveat of the clinical data set that is inconsistent with the endocrine treatment modalities used herein, these results support the idea that increased Mcl-1 expression and activity may be clinically meaningful.

HCC1428-LTED, MCF7-LTED, and T47D-LTED cells were treated with MCL1 si-NPs (or LUC si-NPs, as a control), in the presence or absence of fulvestrant to assess the impact of Mcl-1 in treatment response (or resistance) to the second-line anti-estrogen fulvestrant in cells already resistant to estrogen deprivation. Similar to what was seen in parental HCC1428 and MCF7 cells, Mcl-1 protein expression was upregulated in HCC1428-LTED and MCF-LTED in response to fulvestrant (Fig. 6b). However, MCL1 si-NPs resulted in nearly complete knockdown of Mcl-1 protein expression, negating the ability of cells to upregulate Mcl-1 in response to fulvestrant. As previously reported by other groups, HCC1428-LTED, MCF7-LTED, and T47D-LTED retain their sensitivity to fulvestrant, as demonstrated by modestly increased caspase-3/7 activity (Fig. 6c) and substantially reduced cell growth in monolayer colony assays (Fig. 6d). However, MCL1 si-NPs used in combination with fulvestrant increased caspase-3/7 activity to a greater extent than fulvestrant alone (Fig. 6c) and decreased colony formation to a greater extent than fulvestrant alone (Fig. 6d), confirming that LTED cells, which already use Mcl-1 upregulation to support cell survival, can further increase Mcl-1 in response to additional pressure placed on the ERα pathway by fulvestrant, thus reinforcing their reliance upon Mcl-1 for cell survival^39^. These findings support the feasibility of therapeutic Mcl-1 gene ablation in combination with anti-estrogens^39^ (Fig. 6e).

## Discussion

Previous preclinical studies show that anti-apoptotic Bcl-2 family proteins promote resistance to standard of care breast cancer therapies, including endocrine inhibitors^13–16^, HER2 inhibitors^40^, and chemotherapies^41–43^. An impactful study using preclinical models of ERα+ breast cancers showed extreme sensitive to the combination of tamoxifen and the Bcl-2/Bcl-xL inhibitor ABT-737 or the Bcl-2 specific inhibitor ABT-199, suggesting that Bcl-2 drives resistance of ERα+ breast cancers to tamoxifen^13^. Based on these and other studies, clinical testing of ABT-199 in combination with tamoxifen has begun^44^. However, in a separate study, Bcl-2 expression was decreased in MCF7 xenografts upon tamoxifen treatment and decreased further upon acquisition of tamoxifen resistance^21^, consistent with the clinical finding that Bcl-2 expression often predicts a favorable prognosis^45^, and with the idea that Bcl-2 is a transcriptional target of ERα. These conflicting reports, in addition to a lack of studies observing the role of other anti-apoptotic factors in ERα+ breast cancer resistance, led us to further explore the role of Bcl-2, as well as Bcl-xL and Mcl-1, in response of ERα+ breast cancers to endocrine inhibition.

We found that LTED cells were not sensitive to a physiologically relevant dose of ABT-263, a Bcl-2/Bcl-xL inhibitor that functions similarly to ABT-737. ABT-263 induced tumor cell killing transiently in only one of three LTED-selected ERα+ breast cancer cell lines tested, but this response was not sustained, and ABT-263 did not decrease tumor cell growth in culture or in vivo (Fig. 1e–h). Interestingly, short-term estrogen deprivation (0–48 h) increased Bcl-2 and/or Bcl-xL levels in some, but not all, ERα+ cell lines (Supplemental Figure S1A), although sustained estrogen deprivation caused Bcl-2 and Bcl-xL levels to decrease substantially (Fig. 1c). It is possible that short-term treatment (0–48 h) with tamoxifen or AIs increases Bcl-2 and/or Bcl-xL levels, priming ERα+ breast tumor cells for Bcl-2/Bcl-xL inhibition, as was seen in ERα+ patient-derived xenograft models^13^. However, upon acquisition of resistance, Bcl-2/Bcl-xL levels become depleted, and survival then depends on different anti-apoptotic factors. These ideas will require further exploration in future studies.

Transient sensitivity to ABT-263, as seen in HCC1428-LTED cells, was previously explored in ERα+ breast cancer cell lines grown in estrogen replete conditions^46^. These studies showed that Mcl-1, a Bcl-2-related anti-apoptotic factor, is a key driver of ABT-263 resistance, supporting the concept of compensatory and overlapping functions across anti-apoptotic Bcl-2 family members. Therefore, we were motivated to explore the role of Mcl-1 in our LTED models that were not only resistant to estrogen deprivation, but also to ABT-263. We found abundant Mcl-1 expression (but not Bcl-2 or Bcl-xL) upon LTED in cell culture and in vivo (Figs. 1c and 2a,b), suggesting that Mcl-1, rather than Bcl-2 or Bcl-xL, may be the primary survival factor in AI-resistant ERα+ breast cancers.

Previous reports demonstrate that fulvestrant treatment of ERα+ breast cancer cells causes mRNA upregulation of the pro-apoptotic BH3-only family member, *BIK*, but not other BH3-only factors, such as *PUMA*^33, 34^. Consistent with these reports, we found that fulvestrant-treated HCC1428, MCF7, and T47D cells also upregulated *BIK* mRNA, while *PUMA* levels remained unchanged (Supplemental Figure S5). Despite increased expression of *BIK*, fulvestrant treatment of HCC1428, MCF7, and T47D cells resulted in only modest induction of caspase-3/7 activity in HCC1428 and MCF7 cells, and did not impact caspase-3/7 activity in T47D cells (Fig. 3c)^14^. Interestingly, we found increased Mcl-1/Bim interactions in fulvestrant-treated MCF7 xenografts (Fig. 3d,e), and in clinical ERα+ tumor specimens treated with Faslodex/Arimidex (Fig. 6a). These findings support previous reports showing increased *BIK* expression upon short-term estrogen deprivation and acute fulvestrant treatment^33, 34^, demonstrating that Bik mediates endocrine inhibitor-induced apoptosis. However, our data suggest that upon treatment resistance increased pro-apoptotic signals are thwarted by Mcl-1 upregulation, thus promoting tumor cell survival.

Although previous reports demonstrate that Mcl-1 is a transcriptional target of ERα^47^, data presented herein show that multiple endocrine inhibitors increase Mcl-1 expression and activity in cell culture, in vivo, and in clinical samples, suggesting that ERα may not be a dominant transcriptional activator of Mcl-1. One possibility is that ERα binding sites within the Mcl-1 promoter may repress Mcl-1 gene expression, although this hypothesis has not been tested. Alternatively, the process of Mcl-1 regulation may be very complex, such that ERα inhibition using estrogen deprivation or fulvestrant may change other ERα-regulated transcriptional programs that influence Mcl-1 gene regulation in an indirect manner. These interesting hypotheses will require further investigation, as *MCL1* gene regulation is likely a key deciding element governing tumor cell death in therapeutically treated ERα+ breast cancers.

Since high Mcl-1 activity is known to promote resistance to ABT-263 in breast cancers^12, 32, 48^, these findings suggest that LTED-mediated Mcl-1 induction may indirectly increase resistance to Bcl-2/Bcl-xL inhibition. Thus, it became imperative to inhibit Mcl-1 in combination with Bcl-2/Bcl-xL in LTED cell lines. Mcl-1 inhibition was achieved using nanoparticles loaded with Mcl-1 siRNA (MCL1 si-NPs). The combination of ABT-263 with MCL1 si-NPs increased tumor cell killing and diminished tumor cell growth in each LTED cell line tested (Fig. 4b,c). These findings support the idea that inhibition of multiple anti-apoptotic Bcl-2 family proteins may be required to maximize tumor cell killing. However, a pan-Bcl-2 family inhibitor would have to overcome the challenges of clinical toxicities. For instance, ABT-263 was previously suspended from clinical trials due to extreme thrombocytopenia^49, 50^. Further, it is possible that Mcl-1 targeting alone will be toxic, because Mcl-1 is essential to the survival of normal tissues, such as the heart^51^, brain^52, 53^, and the immune compartment^54–56^. For this reason, we explored Mcl-1 targeting using siRNA, which offers a genetically selective, and possibly less toxic, method to deplete Mcl-1. The nanoparticles into which siRNA sequences were loaded have been vetted for toxicity, and are optimized for superior systemic in vivo delivery over what can be achieved using commercially available reagents. Further, si-NPs penetrate less into healthy tissues as compared to small molecule inhibitors, thus increasing tumor-specific delivery via the enhanced permeability and retention effect^22, 23, 57^, a term describing the leaky vasculature of tumors caused by rapid and irregular tumor angiogenesis. Based on their relative size, si-NPs passively enter and accumulate in tumors^58^, limiting extra-tumoral side effects. Due to these favorable pharmacokinetics, many nanoparticle-based drugs, including siRNA-loaded particles, are in various stages of clinical development [as reviewed in ref.^59^].

Although si-NPs targeting Mcl-1 remain to be tested in vivo, Mcl-1-specific inhibition in cell culture-based models dramatically reduced Mcl-1 expression in both LTED and parental breast cancer cells (Figs. 4a and  5b), increasing tumor cell killing in cells that have developed resistance to current standard of care therapies. Further, we tested the utility of MCL1 si-NPs in combination with fulvestrant, because Mcl-1 expression was induced upon short-term fulvestrant treatment in culture and in vivo (Fig. 3b, d), and tumor cell death was increased upon combined treatment with fulvestrant and MCL1 si-NPs (Fig. 5c). Additionally, fulvestrant treatment further increased tumor cell killing induced by MCL1 si-NPs in LTED-selected cells lines (Fig. 6c), suggesting that Mcl-1 targeting in combination with second-line fulvestrant treatment may be a clinical strategy to increase tumor cell death in ERα+ breast cancer patients. Overall, these studies demonstrate ERα+ breast cancers treated with standard of care endocrine inhibitors may induce Mcl-1 expression and/or activity, which could represent a potential dynamic biomarker of sensitivity to Mcl-1 inhibition. Further, inhibition of Mcl-1 in tumors that are primed by endocrine inhibition may be viable strategy to increase tumor cell killing and breast cancer patient survival.

## Materials and methods

### Cell culture

All cells lines were purchased from the American Tissue Type Collection (*Homo sapiens* ATCC CRL 2327; HTB-22; HTB-133). Cells were maintained in normal growth media (DMEM - Dulbecco's Modified Eagle Medium, 10% fetal bovine serum, 1× antibiotics/anti-mycotics). To generate LTED cells, each cell line was cultured in LTED growth media (phenol-red free Opti-Mem, 10% charcoal stripped fetal bovine serum, 1× antibiotics/anti-mycotics) until they grew at a similar rate to parental cells, 3–6 months. Growth in monolayer (crystal violet) and cell count analyses were completed as in ref.^12^. In cases where parental cell lines were treated with or without estrogen, estrogen was withdrawn after cells were seeded for the corresponding experiment and were cultured in LTED growth media supplemented with or without 2.0 nm 17β-estradiol (Sigma). Otherwise, parental cells were grown and maintained in the presence of estrogen. Fulvestrant (Selleckchem) and ABT-263 (Selleckchem) were dissolved in dimethyl sulfoxide (DMSO) and used at a final concentration of 1.0 µm.

### Caspase-3/7 activity

Five thousand cells/well were plated in 96-well plates in growth media or LTED growth media, and treated with 1.0 μm ABT-263. For experiments with si-NPs, 5000 cells/well (parental cells) or 10,000 cells/well (LTED-selected cells) were plated in 96-well plates and treated with si-NPs for 24 h when growth media was replenished ± 1.0 μm fulvestrant. Caspase activity was determined following the Capsase 3/7-Glo assay (Promega) according to the manufacturer’s instructions at 4 h after adding ABT-263 or 48 h after adding si-NPs. Overall luminescence was read on a Glomax Mutli+ Detection System luminometer (Promega).

### Immunoprecipitation and western analysis

Ten micrograms Bcl-2 (DAKO) or IgG control (Santa Cruz Biotechnologies) antibodies were crosslinked to 50 µL protein A/G agarose beads (Santa Cruz Biotechnologies) using 5 mm BS3 (Sigma-Aldrich) by rotating at room temperature for 30 min. BS3 was quenched using 15 µL 1.0 m Tris (pH 7.4), and beads were washed 3× with one-fourth Nonident-P40 (NP-40) lysis bufferNLB (see below) diluted in 1× PBS. 10 μL crosslinked beads (5 µg antibody) were rotated overnight at 4 °C with 1000 μg protein lysate, harvested in 0.1% Nonident-P40 (NP-40) lysis buffer (NLB) supplemented with protease inhibitor cocktail (Roche), proteasome inhibitor MG-132 (Selleckchem), and sodium orthovanadate (Pierce) as described in ref.^12^. Samples were washed with NLB, boiled in 1× reducing sample buffer (NuPAGE, Invitrogen) and run on a 4–12% sodium dodecyl sulfate polyacrylamide (SDS-PAGE) gel as detailed in ref.^12^. Proteins were transferred to nitrocellulose (iBlot, Invitrogen), blocked 3% gelatin in Tris buffered saline, 0.1% Tween-20 (Sigma-Aldrich), and 3% cold fish gelatin (Sigma-Aldrich), then probed with antibodies against Bim (Cell Signaling Technology, 1:500), Mcl-1 (Santa Cruz Biotechnologies, 1:500), Bcl-xL (Santa Cruz Biotechnologies, 1:500), Actin (Cell Signaling, 1:10,000) and Bcl-2 (DAKO, 1:1000).

### Mice

Mice were housed under pathogen-free conditions and all experiments were in accordance with AAALAC guidelines and with Vanderbilt University Institutional Animal Care and Use Committee approval. MCF7 and MCF7-LTED xenografts were generated as described previously^60^. Tumor volume was measured every other day once tumors became palpable. Treatment of mice began when tumors reached 200 mm^3^. Mice were randomized and treated with 50 µL vehicle control (0.1% Tween 80 and 0.5% methylcellulose) or ABT-263 (20 mg/kg, dissolved in vehicle control) daily by gavage for 16 days or until tumor volume reached 1000 mm^3^. Tissues were collected 1 h after final treatment. MCF7 xenografts were similarly generated and treated with fulvestrant as described in ref.^60^.

### Histological analyses

Tumors were fixed in 10% formalin. Formalin-fixed, paraffin-embedded tumor sections were assessed by terminal dUTP nick end labeling (TUNEL) analysis using the In Situ TUNEL detection kit (source) and for Mcl-1 using methods described in ref.^41^ and anti-Mcl-1 antibody (Santa Cruz Biotechnologies).

### Proximity ligation assay

Formalin-fixed paraffin-embedded sections of human breast tumor biopsies collected as part of a single-stage, single institution phase II neoadjuvant trial, in which post-menopausal women with newly diagnosed breast cancer were treated with anastrozole (1 mg daily, Arimidex) and fulvestrant (250 mg, once per month, Faslodex). After a baseline tumor core biopsy on day 0, patients received treatment (Faslodex and Arimidex). In some cases, patients received the epidermal growth factor receptor (EGFR) kinase inhibitor gefitinib (Iressa)^37, 38^. Tissues were rehydrated using ethanol. Following antigen retrieval using citric acid, the Duolink (Sigma) PLA kit was used according to the manufacturer’s directions using Mcl-1 (Santa Cruz Biotechnologies, 1:25) and Bim (Santa Cruz Biotechnologies, 1:25) antibodies. A Duolink DAPI mounting media was used to counter stain cells. Images were taken at 63× and total puncta/nuclei were counted per field using the ImageJ (Windows 64) Software.

### siRNA-loaded nanoparticles

All polymers were synthesized and characterized as described previously^36^. Two polymers (p(DMAEMA-co-BMA), DB) and (PEG-b-p(BMA-co-DMAEMA), PDB) were used to form si-NPs. Both polymers were dissolved in a 10 mm citric acid buffer (pH 4.0). The siRNA (50 µm in diH_2_O) was initially complexed at 4:1 N:P ratio (ratio of polymer amines:siRNA backbone phosphates) with the DB polymer (0.5 mg/mL polymer concentration) for 15 min at room temperature. Then, PDB polymer (3.33 mg/mL polymer concentration) was added to the mixture to achieve a final N:P ratio of 12:1 and incubated for 30 min at room temperature. Fivefold excess of 10 mm phosphate buffer (pH 8.0) was added to the samples before diluting in fivefold excess of respective growth media. A final siRNA concentration of 100 nm was used to treat cells.

### Statistics

Data presentation and statistics were generated using GraphPad Prism 6 software. Significance was determined by analysis of variance (ANOVA) with Bonferroni post-hoc tests followed by Student’s unpaired two-tailed *t*-test for experiments with more than two conditions, or a Student’s unpaired two-tailed *t*-test for experiments with only two conditions (*P* ≥ 0.05). For tumor growth curves, significance was determined by area under the curve.

## Electronic supplementary material

Supplemental FIgures S1-S5
