# Supplementary material for: Intrinsic apoptotic pathway activation increases response to anti-estrogens in luminal breast cancers
Source: Cell Death Dis. 2018 Jan 17;9(2):21. doi: 10.1038/s41419-017-0072-x (PMC5833697; doi:10.1038/s41419-017-0072-x)
Supplement: Supplementary file 1 — Supplemental FIgures S1-S5 [file 41419_2017_72_MOESM1_ESM.pdf]

A

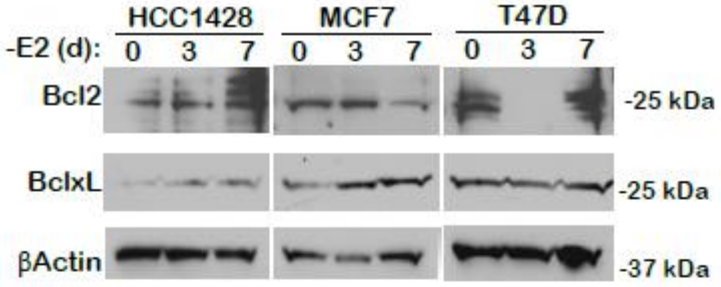

B

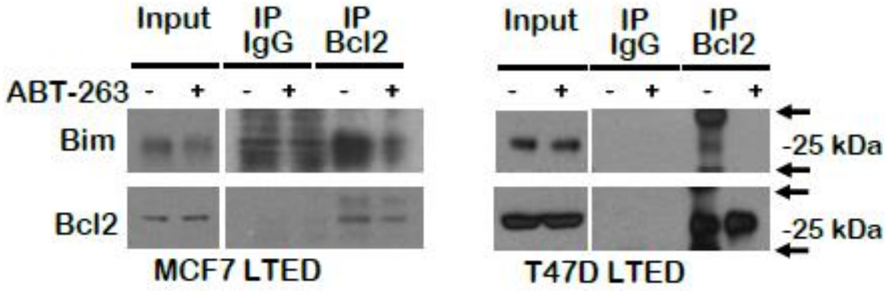

Supplemental Figure S1.

A. Whole cell lysates from luminal breast cancer cell lines grown in estrogen containing conditions (0 d) and estrogen depleted media for 3 and 7 d.

B. Immunoprecipitation completed on whole cell lysates after 2 hrs treatment with ABT-263 (1.0 μM). Arrows indicate background from IgG heavy chain (~50kDa) and IgG light chain (~22kDa).

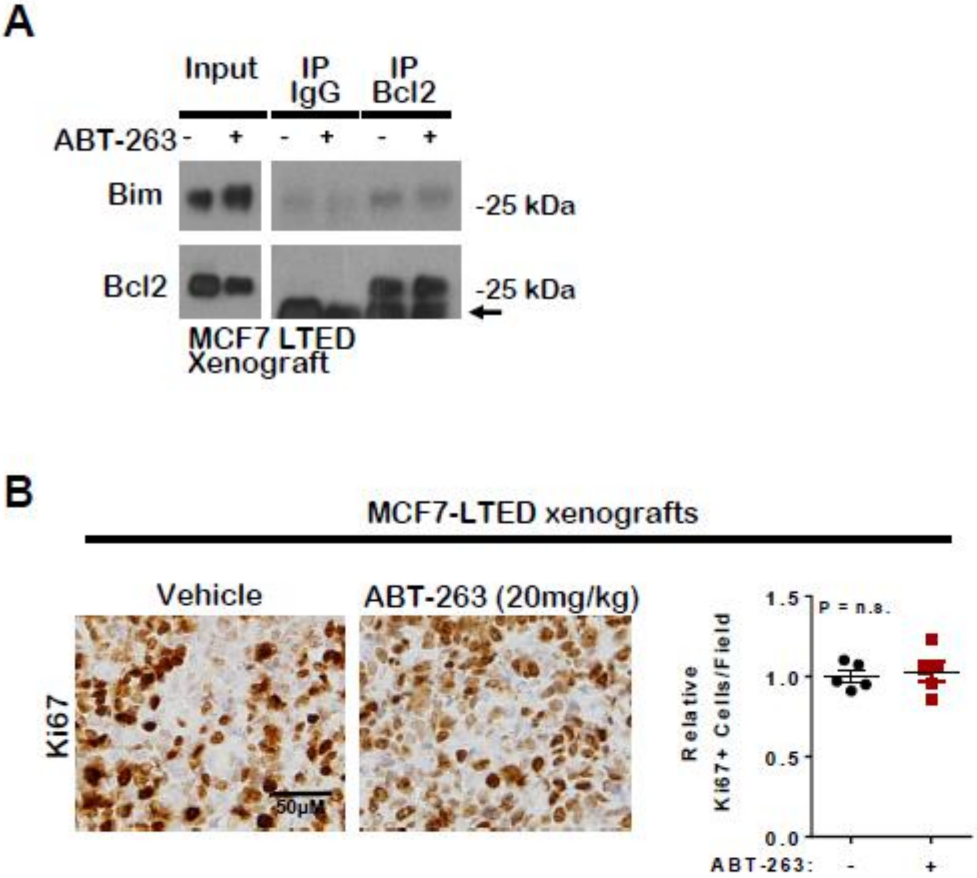

Supplemental Figure S2.

A-B. MCF7 LTED xenografts treated 16 d with ABT-263 (20 mg/kg) or vehicle control.

A. Immunoprecipitation on whole tumor lysates. Arrow points to IgG light chain (~22 kDa).

B. Ki67 staining to demark proliferating cells. Left = representative images, right = quanti tation of average Ki67+ cells/field for 6 fields per tumor. N = 5. Student's unpaired two-tailed t- test, error bars represent standard error.

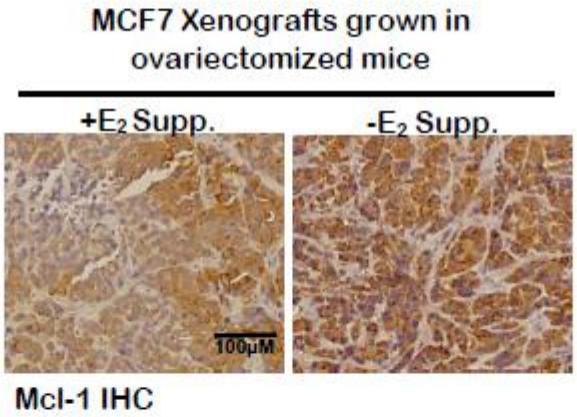

Supplemental Figure S3.

Representative images for Mcl-1 expression in MCF7 LTED xenografts grown in ovariectomized mice ± Estrogen (E_2) supplement (100x).

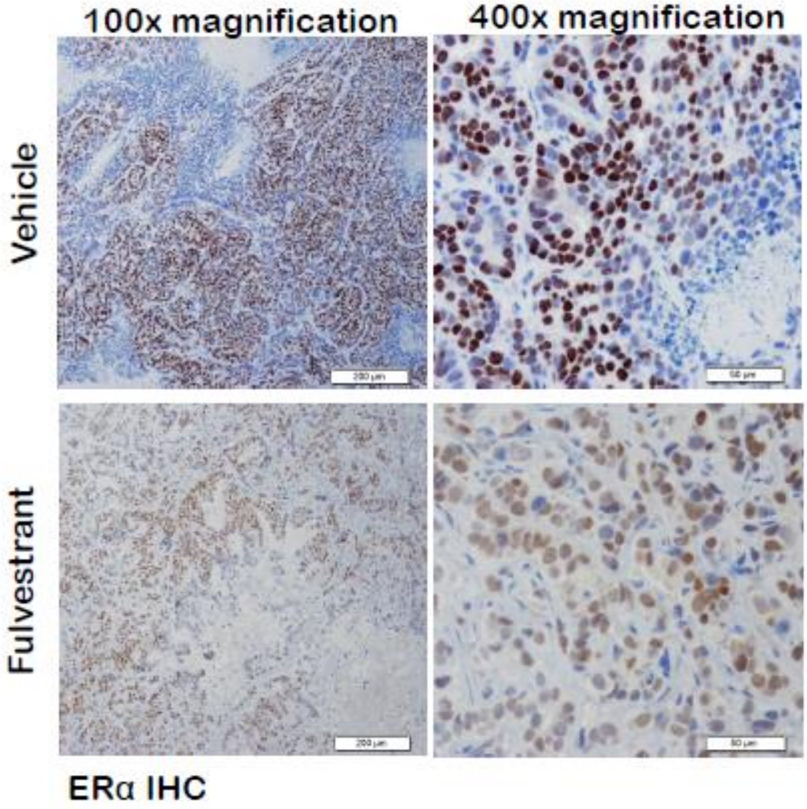

Supplemental Figure 4.

ERα immunohistochemistry performed on sections of MCF7 xenografts treated for 7 days with vehicle or fulvestrant (once weekly by i.p. injection). Representative images taken at 100x and 400x are shown.
